# Redox potentials of ubiquinone, menaquinone, phylloquinone, and plastoquinone in aqueous solution

**DOI:** 10.1007/s11120-017-0433-4

**Published:** 2017-08-22

**Authors:** Shinnosuke Kishi, Keisuke Saito, Yuki Kato, Hiroshi Ishikita

**Affiliations:** 1 Department of Applied Chemistry, The University of Tokyo, 7-3-1 Hongo, Bunkyo-ku, Tokyo, 113-8654 Japan; 2 Research Center for Advanced Science and Technology, The University of Tokyo, 4-6-1 Komaba, Meguro-ku, Tokyo, 153-8904 Japan; 3 Division of Material Science, Graduate School of Science, Nagoya University, Furo-cho, Chikusa-ku, Nagoya, 464-8602 Japan

**Keywords:** Photosystem II, Bacterial photosynthetic reaction centers, *Rhodobacter sphaeroides*, *Blastochloris viridis*, Cytochrome *b*_6_*f*, Cytochrome *bc*_1_

## Abstract

Quinones serve as redox active cofactors in bacterial photosynthetic reaction centers: photosystem I, photosystem II, cytochrome *bc*_1_, and cytochrome *b*_6_ *f*. In particular, ubiquinone is ubiquitous in animals and most bacteria and plays a key role in several cellular processes, e.g., mitochondrial electron transport. Their experimentally measured redox potential values for one-electron reduction *E*_m_(Q/Q^·−^) were already reported in dimethylformamide (DMF) versus saturated calomel electrode but not in water versus normal hydrogen electrode (NHE). We calculated *E*_m_(Q/Q^·−^) of 1,4-quinones using a quantum chemical approach. The calculated energy differences of reduction of Q to Q^·−^ in DMF and water for 1,4-quinone derivatives correlated highly with the experimentally measured *E*_m_(Q/Q^·−^) in DMF and water, respectively. *E*_m_(Q/Q^·−^) were calculated to be −163 mV for ubiquinone, −260 mV for menaquinone and phylloquinone, and −154 mV for plastoquinone in water versus NHE.

## Introduction

**Fig. 1:**
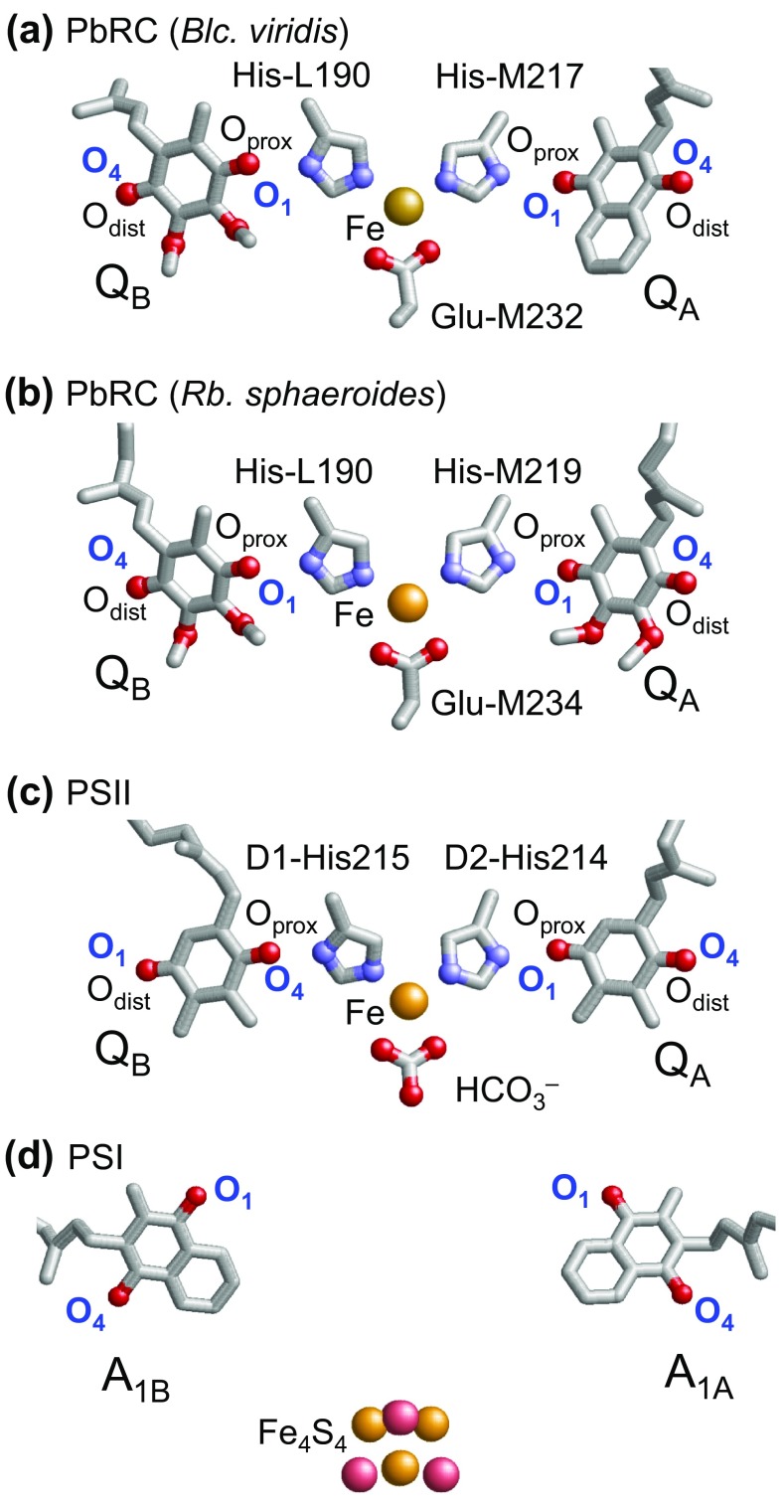
Quinones in photosynthetic reaction centers: **a** menaquinone as Q_A_ and ubiquinone as Q_B_ in bacterial photosynthetic reaction centers from *Blastochloris viridis* (*Blc. viridis*, PDB ID: 2I5N) (Li et al. 2006), **b** ubiquinone as Q_A_ and Q_B_ in bacterial photosynthetic reaction centers from *Rhodobacter sphaeroides* (*Rb. sphaeroides*, PDB ID: 3I4D), **c** plastoquinone as Q_A_ and Q_B_ in PSII (PDB ID: 3ARC) (Umena et al. 2011), and **d** phylloquinone as A_1A_ and A_1B_ in PSI (PDB ID: 1JB0) (Jordan et al. 2001). *Red* and *blue balls* indicate O and N atoms, respectively. In PbRC and PSII, O_prox_ and O_dist_ stand for O atoms of the quinones at the proximal and distal positions with respect to the non-heme Fe complex, respectively. Note that except for Q_B_ in PSII, O_prox_ is O1 and O_dist_ is O4 in PbRC and PSII

**Fig. 2:**
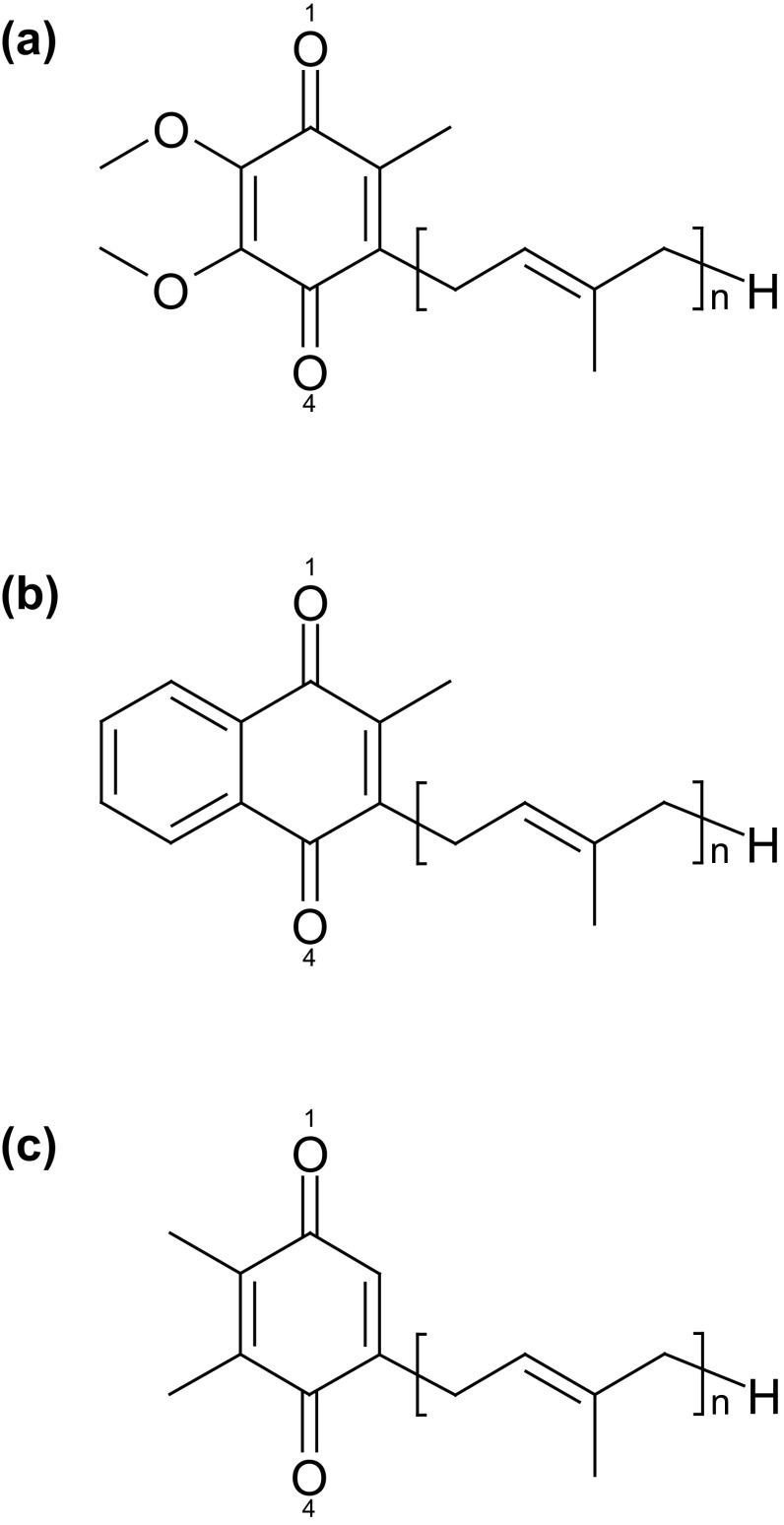
Molecular structures of **a** ubiquinone (*n* = 10), **b** menaquinone and phylloquinone (*n* = 3 to 9), and **c** plastoquinone (*n* = 6 to 9), where *n* is the number of isoprene units

Quinones can accept two electrons and two protons via the initial protonation of semiquinone (Q^·−^ to QH^·^) and the second protonation of hydroquinone (QH^−^ to QH_2_). Ubiquinone serves as an electron acceptor at the Q_A_ and Q_B_ binding sites in reaction centers of purple bacteria (PbRC) from *Rhodobacter sphaeroides* and serves as an electron donor in cytochrome *bc*_1_. Similarly, menaquinone (vitamin K_2_) is the acceptor at the Q_A_ site in PbRC from *Blastochloris viridis*, whereas phylloquinone (vitamin K_1_) is the active center at the A_1A_ and A_1B_ sites in photosystem I (PSI). In reaction centers of green non-sulfur bacteria from *Chloroflexus aurantiacus*, menaquinones are also located at both Q_A_ and Q_B_ sites (Hale et al. 1983). It should be noted that phylloquinone and menaquinone have the same head-group structure (Fig. 2). Plastoquinone serves as an electron acceptor at the Q_A_ and Q_B_ sites in photosystem II (PSII) (Fig. 1) (Robinson and Crofts 1984; Rutherford et al. 1984; Okamura et al. 2000; Brettel and Leibl 2001; Wraight 2004) and serves as an electron donor in cytochrome *b*_6_ *f*. In PbRC and PSII, both Q_A_ and Q_B_ are located near the non-heme Fe^2+^, and the Fe^2+^ ligands (i.e., His-L190 and His-M217 (or M219) in PbRC and D1-His215 and D2-His214 in PSII) donate an H-bond to the carbonyl O atoms of quinones that are proximal to the Fe complex (O_prox_) (Fig. 1a–c). The carbonyl O atoms of quinones at the distal position (O_dist_) also form H-bonds with the proteins. On the other hand, the non-heme Fe^2+^ is absent in PSI, but the Fe_4_S_4_ cluster F_X_ is located near the two A_1_ binding sites (Fig. 1d).

Redox potential values for one-electron reduction, *E*_m_(Q/Q^·−^), for 1,4-quinones, including ubiquinone, menaquinone (phylloquinone), and plastoquinone, were experimentally measured in dimethylformamide (DMF) versus saturated calomel electrode (SCE) by Prince et al. (Prince et al. 1983). *E*_m_(Q/Q^·−^) for 1,4-quinones were also experimentally measured in water versus normal hydrogen electrode (NHE) by Swallow (1982). Since *E*_m_ values for redox active sites in proteins are often reported as the values measured in water versus NHE, *E*_m_(Q/Q^·−^) for ubiquinone, menaquinone (phylloquinone), and plastoquinone measured in water versus NHE are preferentially required when analyzing interaction between the quinone binding site and the protein environment in PbRC, PSI, PSII, cytochrome *bc*_1_, and cytochrome *b*_6_ *f*. However, as far as we are aware, experimentally measured *E*_m_(Q/Q^·−^) for ubiquinone, menaquinone (phylloquinone), and plastoquinone in water versus NHE have not been reported (Fig. 2). Here, we report *E*_m_(Q/Q^·−^) for ubiquinone, menaquinone (phylloquinone), and plastoquinone in water versus NHE, obtained using a quantum chemical approach.

## Computational procedures

In reduction of the oxidized state (A) to reduced state (A^·−^) in aqueous solution, the redox potential *E*_m_ relative to the normal hydrogen electrode (NHE) is defined as

1 $${E_{\text{m}}}=-\frac{{\Delta {G_{{\text{aq}}}}}}{{nF}},$$

where *ΔG*_aq_ is the free energy difference between A and A^·−^ [i.e., *ΔG*_aq_ = *G*_aq_(A^·−^) − *G*_aq_(A) − *G*_NHE_], *n* is the number of electron involved in the reaction (i.e., *n* = 1 in the present case), and *F* is the Faraday constant. *ΔG*_aq_ can also be approximated as

2 $$\Delta{G_{{\text{aq}}}}=k\Delta {E_{{\text{QM/PCM}}}}+C,$$

where *k* is the scaling factor, *ΔE*_QM/PCM_ is the energy difference between A and A^·−^ in aqueous phase [i.e., *ΔE*_QM/PCM_ = *E*_QM/PCM_(A^·−^) − *E*_QM/PCM_(A)], which can be calculated using a quantum chemical approach with the polarizable continuum model (PCM) method, and *C* is a constant (Matsui et al. 2012; Hasegawa et al. 2017). The Eq. 1 can be written as Eq. 3 using Eq. 2,

3 $${E_{\text{m}}}=k^{\prime}\Delta {E_{{\text{QM}}/{\text{PCM}}}}+C^{\prime},$$

where *k′* is the scaling factor and *C′* is a constant (Matsui et al. 2012; Hasegawa et al. 2017). To determine *k′* and *C′*, we calculated *ΔE*_QM/PCM(DMF)_ (and *ΔE*_QM/PCM(water)_) for ten (nine) 1,4-quinones whose experimentally measured *E*_m_(Q/Q^·−^) are reported for DMF (Prince et al. 1983) [and water (Swallow 1982)].

We employed the unrestricted density functional theory (DFT) method with the B3LYP functional and 6-31g++** basis sets for Q^·−^ (the total spin *S* = 1/2) and the restricted DFT method for Q (*S* = 0), using the Gaussian (Frisch et al. 2004) program code with the PCM method. Solvent molecules were considered implicitly, using the SCRF = water option and the SCRF = Dimethylformamide option with the values of 78.3553 for water and 37.219 for DMF for dielectric constant (i.e., default values), respectively. However, it should be noted that it is only one of many internal parameters used to define solvents in the PCM method (Frisch et al. 2004). Thus, simply changing the dielectric constant value will not define a new solvent properly.

Since the isoprene units do not comprised conjugated double bonds, the isoprene side-chain length *n* (Fig. 2) was set to 1 or 2 for the calculations of ubiquinone, menaquinone (phylloquinone), and plastoquinone similar to previous studies (Hasegawa et al. 2017). This could also reduce the number of possible conformations. In fact, the length of the ubiquinone does not practically affect its energetics, as demonstrated by the similar experimentally measured *E*_m_(Q/Q^·−^) values of ubiquinone-1 and -10 in DMF (−611 and −602 mV versus SCE, respectively) (Prince et al. 1983). It should also be noted that *n* = 0, which corresponds to 2,3-dimethoxy-5-methyl-1,4-benzoquinone [*E*_m_(Q/Q^·−^) = −539 mV in DMF versus SCE (Prince et al. 1983)], i.e., ubiquinone-0, as presented in Ref. (Cape et al. 2006), is a less relevant representation of *E*_m_(Q/Q^·−^) for quinones in PbRC, PSI, PSII, cytochrome *bc*_1_, and cytochrome *b*_6_ *f*. Ubiquinone-0 corresponds to 2,3-dimethoxy-5-methyl-1,4-benzoquinone rather than 2,3-dimethoxy-5,6-dimethyl-1,4-benzoquinone [in contrast to the statement in ref. (Prince et al. 1983)].

## Results and discussion

### Correlation of calculated energies with experimentally measured *E*_m_(Q/Q^·−^) for 1,4-quinones in DMF and water

**Fig. 3:**
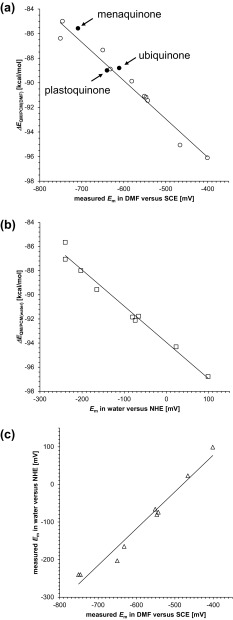
**a** Correlation between experimentally measured *E*_m_(Q/Q^·−^) in DMF versus SCE and calculated Δ*E*_QM/PCM(DMF)_ (coefficient of determination *R* ^2^ = 0.96). Δ*E*_QM/PCM(DMF)_ can be calculated using a quantum chemical approach with the PCM method for DMF. *Closed circles* indicate ubiquinone, menaquinone (phylloquinone), and plastoquinone, whereas *open circles* indicate the other ten 1,4-quinones listed in Table 1 (Prince et al. 1983). The *solid line* was drawn according to Eq. 4 for the ten 1,4-quinones. **b** Correlation between experimentally measured *E*_m_(Q/Q^·−^) in water versus NHE and calculated *ΔE*_QM/PCM(water)_ (coefficient of determination *R* ^2^ = 0.98). *ΔE*_QM/PCM(water)_ can be calculated using a quantum chemical approach with the PCM method for water. *Open squares* indicate the nine 1,4-quinones listed in Table 1 (Swallow 1982). The *solid line* was drawn according to Eq. 5 for the nine 1,4-quinones. **c** Correlation between experimentally measured *E*_m_(Q/Q^·−^) in DMF versus SCE and the experimentally measured *E*_m_(Q/Q^·−^) in water versus NHE (coefficient of determination *R* ^2^ = 0.97). *Open triangles* indicate the nine 1,4-quinones listed in Table 1 (Swallow 1982; Prince et al. 1983). The *solid line* was drawn according to Eq. 6 for the nine 1,4-quinones

The calculated *ΔE*_QM/PCM_ for reduction of deprotonated Q to Q^·−^ for ten 1,4-quinones in DMF (*ΔE*_QM/PCM(DMF)_) and water (*ΔE*_QM/PCM(water)_) were highly associated with the experimentally measured *E*_m_(Q/Q^·−^) in DMF, ranging from −401 to − 751 mV versus SCE (Prince et al. 1983), and the experimentally measured *E*_m_(Q/Q^·−^) in water, ranging from −240 to 99 mV versus NHE (Swallow 1982), which were best fitted to the following equations (Figs. 3a, b):

**Table 1:** Experimentally measured *E*_m_(Q/Q^·−^) (exp.) versus SCE (Prince et al. 1983; Swallow 1982) and calculated *E*_m_(Q/Q^·−^) (calc.) versus NHE

	*E*_m_ in DMF (vs. SCE)		*E*_m_ in water^a^ (vs. NHE)	
	exp.^b^	calc.	exp.^c^	calc.
1,4-Benzoquinone	−401	−399	99	94
Methyl-1,4-benzoquinone	−466	−432	23	12
2,3-Dimethyl-1,4-benzoquinone	−543	−548	−74	−61
2,5-Dimethyl-1,4-benzoquinone	−551	−559	−66	−72
2,6-Dimethyl-1,4-benzoquinone	−547	−557	−80	−70
Trimethyl-1,4-benzoquinone	−632	−630	−165	−146
Tetramethyl-1,4-benzoquinone ^d^	−751	−710	−240	−230
1,4-Naphtoquinone	−581	−598	n.d.	−114
2-Methyl-1,4-naphtoquinone	−650	−679	−203	−198
2,3-Dimethyl-1,4-naphtoquinone	−746	−755	−240	−276
Ubiquinone-1	−611	−633	n.d.	−163
Menaquinone-1 (phylloquinone-1)	n.d.	−738	n.d.	−260
Menaquinone-2	−709	−736	n.d.	−256
Plastoquinone-1	−640	−626	n.d.	−154

*n.d*. Not determined

^a^pH 7

^b^Ref. (Prince et al. 1983)

^c^Ref. (Swallow 1982)

^d^Duroquinone

4 $${E_{\text{m}}}({\text{Q}}/{{\text{Q}}^{ \cdot -}})\;{\text{in DMF}}\;{\text{versus}}\;{\text{SCE }}\left[ {{\text{mV}}} \right]{\text{ }}={\text{ }}-{\text{32}}.{\text{1 }}(\Delta {E_{{\text{QM}}/{\text{PCM}}\left( {{\text{DMF}}} \right)}}+{\text{ 1}}0{\text{8}}.{\text{54 }}\left[ {{\text{kcal}}/{\text{mol}}} \right])$$

5 $${E_{\text{m}}}({\text{Q}}/{{\text{Q}}^{ \cdot -}})\;{\text{in}}\;{\text{ water}}\;{\text{versus}}\;{\text{NHE }}\left[ {{\text{mV}}} \right]{\text{ }}={\text{ }}-{\text{33}}.{\text{3 }}(\Delta {E_{{\text{QM}}/{\text{PCM}}\left( {{\text{water}}} \right)}}+{\text{ 93}}.{\text{95 }}\left[ {{\text{kcal}}/{\text{mol}}} \right]).$$

Using Eqs. 4 and 5, the calculated *E*_m_(Q/Q^··−^) in DMF versus SCE and *E*_m_(Q/Q^·−^) in water versus NHE for ten and nine 1,4-quinones, respectively, are listed in Table 1. Our results confirm that Eqs. 4 and 5 can reproduce the experimentally measured *E*_m_(Q/Q^·−^) in DMF versus SCE and *E*_m_(Q/Q^·−^) in water versus NHE, respectively. The overall root mean square deviation between the experimentally measured *E*_m_(Q/Q^·−^) in DMF versus SCE and the calculated *E*_m_(Q/Q^·−^) in DMF versus SCE based on Eq. 4 for the ten 1,4-quinones is ±21 mV. The overall root mean square deviation between the experimentally measured *E*_m_(Q/Q^·−^) in water versus NHE and the calculated *E*_m_(Q/Q^·−^) in water versus NHE based on Eq. 5 for the nine 1,4-quinones is ±16 mV. These deviations are sufficiently small with respect to those obtained in other theoretical studies [e.g., ±131 mV (Schmidt am Busch and Knapp 2005)].

Notably, to obtain Eq. 4, *E*_m_(Q/Q^·−^) for ubiquinone, menaquinone (phylloquinone), and plastoquinone in DMF versus SCE were not included [where *k* = −32.1 (mV mol/kcal), *C* = −108.5 (mV), excluding ubiquinone, menaquinone (phylloquinone), and plastoquinone]. Nevertheless, the experimentally measured *E*_m_(Q/Q^·−^) in DMF versus SCE (Prince et al. 1983) and the calculated *ΔE*_QM/PCM(DMF)_ for ubiquinone, menaquinone (phylloquinone), and plastoquinone can also be described by Eq. 4 [where *k* = −31.8 (mV mol/kcal), *C* = −108.8 (mV), including ubiquinone, menaquinone (phylloquinone), and plastoquinone] (Fig. 3a), which demonstrates that *E*_m_(Q/Q^·−^) for ubiquinone, menaquinone (phylloquinone), and plastoquinone can be described accurately by Eqs. 4 and 5.

In contrast to other quantum chemical approaches, e.g., (Schmidt am Busch and Knapp 2005), the present approach neither need to calculate the zero-point vibrational energy and the excess vibrational free energy at 298 K for both Q and Q^·−^ nor optimize the atomic radii of Q/Q^·−^ for the solvation energy. Once *k′* and *C′* are uniquely determined, *E*_m_(Q/Q^·−^) can be accurately calculated based on calculated *ΔE*_QM/PCM_, without considering further details of Q/Q^·−^ and solvent. The strong correlation between experimentally measured *E*_m_(Q/Q^·−^) and calculated *ΔE*_QM/PCM_ (Fig. 3), in turn, suggests that *k′* and *C′* are similar for these 1,4-quinones.

In the present study, solvent molecules were considered implicitly. This treatment is more appropriate to describe H-bonds between quinones and bulk water/solvent molecules, in which the H-bond patterns are not unique, e.g., bulk solvent. Explicit water/solvent models may be able to describe H-bonds adequately when the H-bond pattern is unique [e.g., water molecules in the well-ordered cluster near the Mn_4_CaO_5_ cluster (Saito et al. 2011; Sakashita et al. 2017)] or all possible (H-bond) conformations of water/solvent molecules can be evaluated, e.g., using molecular dynamics simulations; this is not the case for 1,4-quinones investigated in the present study.

We found that the experimentally measured *E*_m_(Q/Q^·−^) for the nine 1,4-quinones in water versus NHE (Swallow 1982) and DMF versus SCE (Prince et al. 1983) correlated strongly (Fig. 3c), which were best fitted to the following equation:

6 $${E_{\text{m}}}({\text{Q}}/{{\text{Q}}^{ \cdot -}})\;{\text{in }}\;{\text{water}}\;{\text{versus}}\;{\text{NHE }}\left[ {{\text{mV}}} \right]{\text{ }}={\text{ }}0.{\text{98 }}[{E_{\text{m}}}({\text{Q}}/{{\text{Q}}^{ \cdot -}}){\text{ in DMF}}\;{\text{versus}}\;{\text{SCE }}+{\text{ 48}}0].$$

Equation 6 indicates that experimentally measured *E*_m_(Q/Q^·−^) in DMF versus SCE can be practically converted to *E*_m_(Q/Q^·−^) in water versus NHE by adding 480 mV. The *E*_m_ difference of 480 mV may also contain a liquid junction potential between SCE in DMF and NHE in water. The liquid junction potential can be ignored when *E*_m_(Q/Q^·−^) are compared versus ferrocene (Fc/Fc^+^); e.g., *E*_m_(Q/Q^·−^) for 1,4-benzoquinone is experimentally measured to be −401 mV in DMF versus SCE, where *E*_m_(Fc/Fc^+^) = 524 mV (Prince et al. 1983). Since *E*_m_(Fc/Fc^+^) = 400 mV in water versus NHE (Koepp et al. 1960), *E*_m_(Q/Q^·−^) for 1,4-benzoquinone is −925 mV in DMF versus Fc/Fc^+^ and 301 mV in water versus Fc/Fc^+^, which indicates that *E*_m_(Q/Q^·−^) for 1,4-benzoquinone in DMF and water originally differ by 624 mV in the absence of the liquid junction potential (Table 2). This holds true for all 1,4-quinones investigated. It seems likely that *E*_m_(Q/Q^·−^) for 1,4-benzoquinones already differ by 600 mV even in the absence of the liquid junction potential (Table 2). The presence of H-bond donor to Q^·−^ in water is partly responsible for the *E*_m_ difference of 600 mV, since the presence of H-bond donor to Q^·−^ stabilizes Q^·−^ and increases *E*_m_(Q/Q^·−^). Nevertheless, the entire difference of 600 mV would not be explained solely by the first sphere water molecules that can directly form an H-bond with Q^·−^. The surrounding water molecules (e.g., second and third sphere molecules) cannot directly form an H-bond with Q^·−^ but the Q^·−^ stabilization is pronounced by their dipole orientations (Takaoka et al. 2016). The corresponding effect may be ignored in DMF with respect to water.

**Table 2:** Experimentally measured *E*_m_(Q/Q^·−^) (exp.) (Prince et al. 1983; Swallow 1982), calculated *E*_m_(Q/Q^·−^) (calc.) versus ferrocene (Fc/Fc^+^), and the difference in *E*_m_(Q/Q^·−^) (*ΔE*_m_)

	*E*_m_ in DMF (vs. Fc/Fc^+^)		*E*_m_ in water^a^ (vs. Fc/Fc^+^)		*ΔE*_m_ (DMF–water)	
	exp.^b^	calc.	exp.^c^	calc.	exp.	calc.
1,4-Benzoquinone	−925	−923	−301	−306	−624	−617
Methyl-1,4-benzoquinone	−990	−956	−377	−388	−613	−568
2,3-Dimethyl-1,4-benzoquinone	−1067	−1072	−474	−461	−593	−611
2,5-Dimethyl-1,4-benzoquinone	−1075	−1083	−466	−472	−609	−611
2,6-Dimethyl-1,4-benzoquinone	−1071	−1081	−480	−470	−591	−611
Trimethyl-1,4-benzoquinone	−1156	−1154	−565	−546	−591	−608
Tetramethyl-1,4-benzoquinone^d^	−1275	−1234	−640	−630	−635	−604
1,4-Naphtoquinone	−1105	−1122	n.d.	−400	n.d.	n.d.
2-Methyl-1,4-naphtoquinone	−1174	−1203	−603	−598	−571	−605
2,3-Dimethyl-1,4-naphtoquinone	−1270	−1279	−640	−676	−630	−602
Ubiquinone-1	−1135	−1157	n.d.	−563	n.d.	−593
Menaquinone-2 (phylloquinone-2)	−1233	−1260	n.d.	−660	n.d.	−600
Plastoquinone-1	−1164	−1150	n.d.	−554	n.d.	−596

*n.d*. Not determined

^a^pH 7

^b^Ref. (Prince et al. 1983)

^c^Ref. (Swallow 1982)

^d^Duroquinone

As far as only *E*_m_ differences among the redox active cofactors (*ΔE*_m_) are discussed in the same proteins, e.g., along the electron transfer chains, *E*_m_ values of isolated cofactors measured in DMF, which are reported also for chlorophylls (Watanabe and Kobayashi 1991), might possibly be useful. On the other hand, when *E*_m_ values in the protein environments are discussed, comparison with *E*_m_ values of isolated cofactors measured in water is recommended, since *E*_m_ values measured in DMF is originally 600 mV lower than those measured in water even in the absence of the liquid junction potential (Table 2).

### *E*_m_(Q/Q^·−^) for ubiquinone, menaquinone, phylloquinone, and plastoquinone in water versus NHE

To the best of our knowledge, experimentally measured *E*_m_(Q/Q^·−^) for ubiquinone, menaquinone (phylloquinone), and plastoquinone in water versus NHE are not reported. By calculating *ΔE*_QM/PCM(water)_ and using Eq. 5, *E*_m_(Q/Q^·−^) was calculated to be −163 mV for ubiquinone, −260 mV for menaquinone (phylloquinone), and −154 mV for plastoquinone in water versus NHE (Table 1).

In ubiquinone, one of the 2,3-methoxy groups lies outside the quinone ring. Hence, Zhu and Gunner proposed that *E*_m_(Q/Q^·−^) for ubiquinone, a 2,3-dimethoxy-5-methyl-6-isoprenyl benzoquinone, is more similar to the *E*_m_(Q/Q^·−^) for trimethyl-benzoquinone than to the *E*_m_(Q/Q^·−^) for tetramethyl-benzoquinone (Zhu and Gunner 2005). Indeed, the calculated *E*_m_(Q/Q^·−^) = −163 mV for ubiquinone (Table 1) is close to the experimentally measured *E*_m_(Q/Q^·−^) = −165 mV for trimethyl-benzoquinone (Swallow 1982) in water versus NHE, which is consistent with their proposal. Although it was proposed that difference in the 2-methoxy orientation of ubiquinone was responsible for the *E*_m_ difference of more than 160 mV between Q_A_ and Q_B_ in PbRC (Taguchi et al. 2013), the similar *E*_m_(Q/Q^·−^) values of trimethyl-benzoquinone and ubiquinone (ref. (Zhu and Gunner 2005) and Table 1) suggest that contributions of methoxy and methyl groups to *E*_m_(Q/Q^·−^) are not significantly different. It should also be noted that estimation by Swallow resulted in a more negative value of *E*_m_(Q/Q^·−^) = −230 ± 20 mV for ubiquinone at pH 7 (Swallow 1982).

The present study shows that *E*_m_(Q/Q^·−^) is −260 mV for menaquinone (phylloquinone) in water versus NHE (Table 1); the calculated *E*_m_(Q/Q^·−^) can be confirmed by Eq. 6, which can be reproduced by adding 480 mV to *E*_m_(Q/Q^·−^) in DMF versus SCE. Previously, Ptushenko et al. considered that *E*_m_(Q/Q^·−^) was −800 mV for phylloquinone in DMF versus NHE by considering a liquid junction potential between SCE in DMF and NHE in water (Ptushenko et al. 2008). Using the low *E*_m_(Q/Q^·−^) value of −800 mV for phylloquinone in DMF versus NHE, they obtained *E*_m_(A_1A_) = −671 mV and *E*_m_(A_1B_) = −844 mV (Ptushenko et al. 2008), and were able to reproduce the reported low *E*_m_(A_1_) in PSI [e.g., −810 mV (Vos and van Gorkom 1990), −754 mV (Iwaki and Itoh 1994), and lower than −700 mV (Brettel and Leibl 2001)]. This, in turn, suggests that the electrostatic interaction of the PSI protein environment at the A_1_ site is remarkably weak in their computational model. If this is the case, then *E*_m_(Q_A_) of −150 mV for the same quinone species (menaquinone) would be regarded as being “unusually high” in PbRC from *Blastochloris viridis* (Brettel and Leibl 2001), and the PbRC protein environment must dramatically increase *E*_m_(Q/Q^·−^) for menaquinone by more than 600 mV at the Q_A_ site in their computational model; obviously this is not the case for the PbRC protein environment, as already demonstrated in theoretical studies (Rabenstein et al. 1998; Ishikita and Knapp 2004; Zhu and Gunner 2005). *E*_m_(Q/Q^·−^) = −260 mV for menaquinone (phylloquinone) in water versus NHE (Table 1) suggests that the PSI protein environment (e.g., the presence of negatively charged F_X_ near A_1_ (Ishikita and Knapp 2003)) is responsible for low *E*_m_(A_1_) in PSI. When *E*_m_(Q/Q^·−^) = −800 mV for phylloquinone in DMF versus NHE is used, the resulting *E*_m_(A_1_) should contain the *E*_m_ downshift of ca. 600 mV with respect to water versus NHE as an artifact (Table 2), since the PSI is not solvated in DMF but in water in the thylakoid membrane. One can directly focus on the influence of the PSI protein environment on *E*_m_(A_1_) when using *E*_m_(Q/Q^·−^) = −260 mV in water versus NHE. It seems plausible that using *E*_m_ values measured in water is more recommended to analyze *E*_m_ values for the redox active groups in proteins unless the proteins are solvated in DMF.

This fact would be more obvious when considering *E*_m_ of heme proteins or flavin-binding proteins. *E*_m_ of heme (Harbury and Loach 1960; Wilson 1983) and flavin (Draper and Ingraham 1968; Anderson 1983) were experimentally measured in water. These cofactors are often largely exposed to the protein bulk surface [e.g., heme (Kerfeld et al. 2003; Clarke et al. 2011) and flavin-binding (Ludwig et al. 1997; Watt et al. 1991) proteins]. As these cofactors are released away from the binding site toward the bulk region, the *E*_m_ values must be close to those experimentally measured in water; this is exactly the case for Q_B_ in PbRC and PSII, which is located near the protein bulk surface. Using spectroelectrochemistry, Kato et al. directly determined *E*_m_(Q_B_) to be +90 mV in PSII from *Thermosynechococcus elongates* versus NHE (Kato et al. 2016). *E*_m_(Q/Q^·−^) is −154 mV for plastoquinone in water versus NHE (Table 1) and would be −750 mV in DMF versus NHE (assuming *E*_m_ downshift of ca. 600 mV, Table 2). If *E*_m_(Q/Q^·−^) measured in DMF were relevant, the PSII protein environment would need to increase *E*_m_(Q/Q^·−^) for plastoquinone by 840 mV at the Q_B_ site. In addition, *E*_m_(Q_A_) was determined to be −145 mV in spinach PSII versus NHE, using spectroelectrochemistry (Brinkert et al. 2016); the PSII protein environment would also need to increase *E*_m_(Q/Q^·−^) for plastoquinone by 600 mV even at the Q_A_ site, which is less exposed to the protein bulk surface. It seems likely that *E*_m_ values for quinones measured in water are more recommended when comparing with *E*_m_(Q/Q^·−^) in the protein environments. This would also hold true for the quinone binding sites in cytochrome *bc*_1_ and cytochrome *b*_6_ *f*, at which quinones from PbRC and PSII can bind, respectively.

## Conclusion

Experimentally measured *E*_m_(Q/Q^·−^) in DMF versus SCE (Prince et al. 1983) and *E*_m_(Q/Q^·−^) in water versus NHE (Swallow 1982) correlated highly with the quantum chemically calculated energy differences (*ΔE*_QM/PCM_) between neutral and reduced states (Figs. 3a, b) and can be best fitted to Eqs. 4 and 5, respectively. It seems likely that *E*_m_(Q/Q^·−^) for 1,4-benzoquinones differ by 600 mV even in the absence of the liquid junction potential between DMF and NHE (versus Fc/Fc^+^). *E*_m_(Q/Q^·−^) was calculated to be −163 mV for ubiquinone, −260 mV for menaquinone (phylloquinone), and −154 mV for plastoquinone in water versus NHE (Table 1). In particular, *E*_m_(Q/Q^·−^) = −260 mV for phylloquinone in water versus NHE unambiguously demonstrates that remarkably low *E*_m_(A_1_) in PSI does not originate from *E*_m_(Q/Q^·−^) for phylloquinone but from interaction with the PSI protein environment, as suggested previously (Ishikita and Knapp 2003). These *E*_m_(Q/Q^·−^) are prerequisite for analyzing the *E*_m_(Q/Q^·−^) shift caused by electrostatic interactions within the protein environment in photosynthetic reaction centers.
